# A novel NET-related gene signature for predicting DLBCL prognosis

**DOI:** 10.1186/s12967-023-04494-9

**Published:** 2023-09-16

**Authors:** Huizhong Shi, Yiming Pan, Guifen Xiang, Mingwei Wang, Yusong Huang, Liu He, Jue Wang, Qian Fang, Ling Li, Zhong Liu

**Affiliations:** 1 Department of Hematology, Shanghai General Hospital, Shanghai Jiao Tong University School of Medicine, No. 100 Haining Road, Shanghai, 200080 China; 2 Institute of Blood Transfusion, Chinese Academy of Medical Sciences and Peking Union Medical College, 26 Huacai Rd, Longtan Industry Zone, Chenghua District, Chengdu, 610052 Sichuan China; 3 Key Laboratory of Transfusion Adverse Reactions, Chinese Academy of Medical Sciences, 26 Huacai Rd, Longtan Industry Zone, Chenghua District, Chengdu, 610052 Sichuan China; 4 School of Public Health, Anhui Medical University, Hefei, 230032 China; 5 Department of Cardiology, Shanghai General Hospital, Shanghai Jiao Tong University School of Medicine, Shanghai, 200080 China; 6 Stomatology Center, Affiliated Hospital of Hangzhou Normal University, Hangzhou, 310000 China; 7 Department of Blood Transfusion, the Third People’s Hospital of Chengdu, Affiliated Hospital of Southwest Jiaotong University, 82 Qinglong Street, Qingyang District, Chengdu, 610031 Sichuan China

**Keywords:** Diffuse large B-cell lymphoma, Neutrophil extracellular traps, Prognostic model, Tumor microenvironment, Prognostic biomarker

## Abstract

**Background:**

Diffuse large B-cell lymphoma (DLBCL) is an aggressive malignancy. Neutrophil extracellular traps (NETs) are pathogen-trapping structures in the tumor microenvironment that affect DLBCL progression. However, the predictive function of NET-related genes (NRGs) in DLBCL has received little attention. This study aimed to investigate the interaction between NRGs and the prognosis of DLBCL as well as their possible association with the immunological microenvironment.

**Methods:**

The gene expression and clinical data of patients with DLBCL were downloaded from the Gene Expression Omnibus database. We identified 148 NRGs through the manual collection of literature. GSE10846 (n = 400, GPL570) was used as the training dataset and divided into training and testing sets in a 7:3 ratio. Univariate Cox regression analysis was used to identify overall survival (OS)-related NETs, and the least absolute shrinkage and selection operator was used to evaluate the predictive efficacy of the NRGs. Kaplan–Meier plots were used to visualize survival functions. Receiver operating characteristic (ROC) curves were used to assess the prognostic predictive ability of NRG-based features. A nomogram containing the clinical information and prognostic scores of the patients was constructed using multivariate logistic regression and Cox proportional risk regression models.

**Results:**

We identified 36 NRGs that significantly affected patient overall survival (OS). Eight NRGs (PARVB, LYZ, PPARGC1A, HIF1A, SPP1, CDH1, S100A9, and CXCL2) were found to have excellent predictive potential for patient survival. For the 1-, 3-, and 5-year survival rates, the obtained areas under the receiver operating characteristic curve values were 0.8, 0.82, and 0.79, respectively. In the training set, patients in the high NRG risk group presented a poorer prognosis (*p* < 0.0001), which was validated using two external datasets (GSE11318 and GSE34171). The calibration curves of the nomogram showed that it had excellent predictive ability. Moreover, in vitro quantitative real-time PCR (qPCR) results showed that the mRNA expression levels of CXCL2, LYZ, and PARVB were significantly higher in the DLBCL group.

**Conclusions:**

We developed a genetic risk model based on NRGs to predict the prognosis of patients with DLBCL, which may assist in the selection of treatment drugs for these patients.

**Supplementary Information:**

The online version contains supplementary material available at 10.1186/s12967-023-04494-9.

## Introduction

Diffuse large B-cell lymphoma (DLBCL) is a common aggressive form of non-Hodgkin's lymphoma. Globally, approximately 150,000 new DLBCL cases are reported annually [1]. Its clinical features and prognoses are highly heterogeneous. The classic international prognostic index (IPI), which classifies patients into four risk groups, is widely used by clinicians as a valid prognostic tool in clinical practice [2]. However, its ability to differentiate between the four risk groups is reduced by the use of rituximab, especially in high-risk patients [3–5]. In addition, due to the high tumor heterogeneity, even patients in the same IPI risk category exhibit different clinical outcomes [6]. Currently, the 5-year survival rate for first-line treatment, as represented by R-CHOP, is 60–70%, with up to 50% of patients presenting as chemo-refractory or relapsing after treatment [7]. Thus, it is vital to develop new markers for evaluating patient prognosis.

Neutrophils, the most abundant immune cells in the bone marrow and peripheral blood, have various functions in cancer development, growth, and metastasis [8]. As part of the inflammatory cells in the tumor microenvironment, neutrophils play both pro- and anticancer roles [9]. In solid tumors, the neutrophil-to-lymphocyte ratio is an independent prognostic indicator for several malignancies [10]. Most clinical evidence supports the notion that neutrophils promote cancer progression in solid tumors [11, 12]. At the same time, cancer cells shape the tumor microenvironment by secreting various cytokines and chemokines, providing the necessary environmental conditions for the reprogramming of neutrophils, which may explain the varied functions of neutrophils in cancer [12]. In DLBCL, tumor-associated neutrophils contribute to the survival, growth, and drug resistance of malignant B cells [13]. Neutrophils not only act through degranulation and phagocytosis but also create neutrophil extracellular traps (NETs), which destroy bacterial virulence factors and kill bacteria [14]. Recent studies have shown that patients with DLBCL who have more NETs in their plasma or tumor tissues have a poorer prognosis. Thus, we hypothesized that NETs could act as new prognostic markers in patients with DLBCL [15].

In this study, we identified the NET-regulated genes (NRGs) associated with survival in patients with DLBCL and developed a prognostic model. NRGs were obtained from previously published studies. DLBCL transcriptomic datasets were downloaded from the Gene Expression Omnibus (GEO) database and divided into training and validation datasets. Univariate Cox regression analysis was performed to assess the prognostic impact of NETs in DLBCL, and the least absolute shrinkage and selection operator (LASSO) Cox regression was used to identify key variables and construct the risk characteristics associated with NRGs. To test predictive performance, we analyzed the predictive accuracy of the prognostic markers on a validation set. The results showed that the risk score was a reliable prognostic indicator independent of other clinical parameters. In this study, we constructed a risk model based on NRGs and explored the unique immune cell infiltration landscape of the tumor microenvironment as well as identified potential drug targets.

## Methods

### Data collection and processing

Three DLBCL transcriptome microarrays (GSE10846, GSE11318, and GSE34171) based on the Affymetrix Human Genome U133 Plus 2.0 Array platform containing clinical and transcriptomic data for 420, 203, and 68 patients with DLBCL, respectively, were downloaded from the GEO database. To increase the accuracy of the study, we used the normalize between arrays function in the R package ‘limma’ (version 3.54.2) to normalize the expression matrix and eliminate patients with a survival time of less than 30 days. We then interpolated the missing values from the clinical data using the R package ‘mice’ (version 3.15.0). We selected GSE10846 (n = 400) as the training dataset And GSE11318 (n = 193) and GSE34171 (n = 67) as the external validation datasets. The clinical information of the patients is listed in Additional file 1: Table S1.

### Acquisition of NET gene list

We identified 148 genes for analysis by manually collecting previously published NRGs from the literature, see Additional file 2: Table S2 for a film showing this in more detail.

### Consensus clustering analysis and principal component analysis (PCA)

The ‘ConsensusClusterPlus’ package (version 1.60.0) was used to set the model to cohesive "means" clustering, utilizing Euclidean correlation distances and 50 replicates for 80% of the samples [16]. The R package ‘FactoMineR’ (version 2.7) was used for PCA analysis and visualization to show the distribution between groups [17].

### Identification of differentially expressed genes (DEGs)

We utilized the R package ‘limma’ (version 3.54.2) to identify DEGs between the two clusters and risk groups. DEGs were determined using the threshold |log2FC|≥ 1 and *p* < 0.05.

### Functional enrichment analysis

We conducted Gene Ontology (GO) and Kyoto Encyclopedia of Genes and Genomes (KEGG) enrichment analyses of the DEGs using the R package ‘clusterProfiler’ (version 4.7.1.3) [18]. Terms with *p* < 0.05 were considered as significantly enriched.

### Identification of overall survival (OS)-related NRGs

The GSE10846 dataset was randomly divided into training and testing sets at a ratio of 7:3. Univariate Cox regression analysis was used to identify OS-related NETs in the training cohort (n = 280; *p* < 0.05).

### Construction and validation of NRG-related prognostic model for patients with DLBCL

We used the R packages ‘glmnet’ (version 4.1.6) and ‘survival’ (version 3.4.0) on the training dataset to identify the most significant features in OS-related NRGs using LASSO Cox regression to avoid overfitting [19]. We then constructed a multivariate Cox proportional risk model based on the Akaike Information Criterion for forward and backward stepwise selection [20]. The risk score of the prognostic signature was calculated as follows:

$$Risk \,score=\sum \limits_{i}^{n}Coe{f}_{i}*{A}_{i}$$

where $Co{ef}_{i}$ is the regression coefficient of the Cox model, $i$ represents the corresponding index of each NRG that comprised of the signature, ${A}_{i}$ represents the relative value of the expression of the individual NRG in the signature, and $n$ represents the number of genes in the signature. The R package ‘survminer’ (version 0.4.9) was used to select the best cutoff value and divide the patients into low- and high-risk groups. Kaplan–Meier survival curves were created using the same package, and further log-rank tests were performed to examine how events unfolded chronologically in each group. Time-dependent receiver operating characteristic (ROC) curves were used to assess the predictive power of the models [21]. We then used Kaplan–Meier survival analysis and log-rank testing to determine patient survival.

To verify the predictive ability of the model, GSE11318 (n = 193) and GSE34171 (n = 67) were used as testing sets. Risk scores were calculated for each patient, and Kaplan–Meier survival curves were used to reflect their performance in OS. The ability of NRG-based features to predict prognosis was assessed using time-dependent receiver operating characteristic (ROC) curves.

### Analysis of the tumor immune microenvironment

For gene expression data in patients with DLBCL, single-sample gene set enrichment analysis (ssGSEA) was performed to estimate the infiltration abundance of 28 different immune cell types [22]. Immune cells with a *p* < 0.05 were considered to be significantly different.

### Correlation analysis of immune checkpoints and risk scores

Recently, immunotherapy and targeted therapies for DLBCL have received increasing attention. Here, we conducted Pearson correlation analysis to examine the association between risk scores and treatment targets in clinical trials and clinical practice to assess the possible impact of risk score-based treatments. Selected targets were PLK1, CD33, DOT1L, CHEK1, CD47, MCL1, ASXL1, IDH1, MDM2, and BCL2.

### Construction of a NET-related clinicopathologic nomogram

The R package ‘rms’ (version 6.5.0) was used to create a NET-related clinicopathological nomogram for predicting the OS of the patients by incorporating the prognostic signature into the clinicopathological characteristics available in the training set [23]. Calibration curves were generated using the R package ‘PredictABEL’ (version 1.2.4) to predict OS in patients with DLBCL.

### Prediction of half-maximal inhibitory concentration (IC50) values for different targeted therapy agents

Based on the gene expression level, the R package ‘oncoPredict’ (version 0.2) was used to predict the IC50 values of the targeted drugs [24].

### Drug-NRGs network analysis

The drug-gene interaction database (DGIdb, https://dgidb.genome.wustl.edu/), an online tool for drug-gene prediction, was used to explore the target drugs of the eight NRGs [25].

### Molecular docking

Molecular docking simulations are used to predict the formation of stable complexes between large and small molecules. The structure of the HIF1A protein was obtained from the Protein Data Bank (https://www.rcsb.org/), and related small molecules were obtained from the PubChem database (https://pubchem.ncbi.nlm.nih.gov) [26]. AutoDockTools and Discovery Studio 2021 client were used to preprocess the input file, including the hydrogenation and deletion of crystallographic water and ligands. Molecular docking between the small molecules and HIF1A binding pockets was carried out using the AutoDock Vina program with default parameters. The predicted binding interaction geometries of HIF1A were visualized, and the docking affinity between small molecules and protein targets was assessed. The optimal docking conformation and related results were analyzed using Discovery Studio 2021.

### Cell lines and cell culture

The DLBCL cell line OCI-LY3 was obtained from Bolsen Biotechnology Company (Shanghai, China). Human blood B lymphocytes (IM-9) were obtained from the Beina Chuanglian Institute of Biotechnology (Beijing, China). OCI-LY3, IM-9 cells were grown in RPMI-1640 (Thermo Fisher Scientific, USA).

### RNA extraction, cDNA synthesis, and quantitative real-time PCR (qPCR)

Total RNA was isolated using TRIzol reagent (Thermo Fisher Scientific). First-strand cDNA was synthesized from total RNA using Transcript All-in-One First-strand cDNA Synthesis SuperMix for qPCR (TransGen, Beijing, China). RT-qPCR was performed using PerfectStart Green qPCR SuperMix on CFX Maestro (Bio-Rad Laboratories, Hercules, CA, USA), with GAPDH as an internal loading control. The Bio-Rad CFX Maestro real-time monitoring system was used according to the manufacturer's instructions. Relative levels were calculated by the relative quantification 2^(-ΔΔCT) method. The mean value of the relative mRNA expression in the control samples was set to 1. Primers were purchased from Sangon Biotechnology Co., Ltd. (Beijing, China), and their sequences are listed in Table 1.

**Table 1:** Primer sequences were used in this study

Genes	Forward primer sequence	Reverse primer sequence
*PARVB*	TGGACTCAATTCACGGGAAGA	GCACCGTTACATGCTCAGGA
*S100A9*	CAACACCTTCCACCAATACTCT	AGGTCCTCCATGATGTGTTCT
*CXCL2*	CCGAAGTCATAGCCACACTCA	TGGATTTGCCATTTTTCAGCATCT
*SPP1*	GAAGTTTCGCAGACCTGACAT	GTATGCACCATTCAACTCCTCG
*LYZ*	GGCCAAATGGGAGAGTGGTTA	CCAGTAGCGGCTATTGATCTGAA
*HIF1A*	ATCCATGTGACCATGAGGAAATG	TCGGCTAGTTAGGGTACACTTC
*CDH1*	ATTTTTCCCTCGACACCCGAT	TCCCAGGCGTAGACCAAGA
*PPARGC1A*	GCTTTCTGGGTGGACTCAAGT	GAGGGCAATCCGTCTTCATCC
*GAPDH*	CTGGGCTACACTGAGCACC	AAGTGGTCGTTGAGGGCAATG

### Statistical analysis

Statistical analyses were conducted using R software (version 4.2.1). DEGs were identified using linear models and the empirical Bayes method. The Spearman correlation coefficient was used for correlation analysis. Differential analysis of continuous variables with a normal or non-normal distribution was performed using the Student's *t-*test or Wilcoxon rank-sum test, respectively. All *p*-values in this study are two-tailed, and statistical significance was set at *p* < 0.05.

## Results

### Analysis of gene function and clustering

**Fig. 1:**
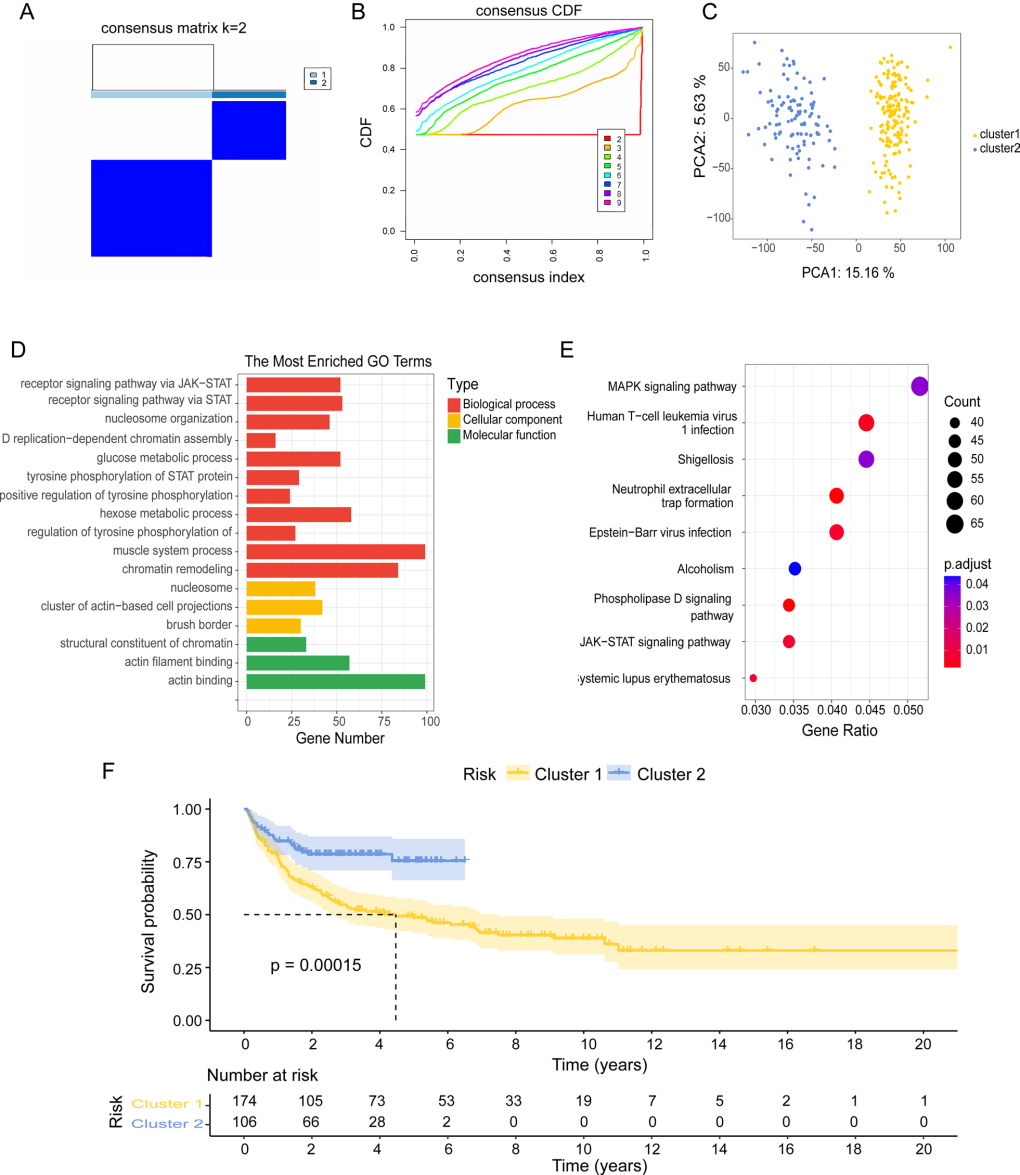
Consensus clustering and functional annotation of DEGs between the two clusters. **A**, **B** Consensus matrix heatmap defining two clusters (k = 2) and their correlation area. **C** PCA showing a clear difference in the transcriptomes between cluster 1 (n = 174) and cluster 2 (n = 106). **D**, **E** GO and KEGG enrichment analyses of DEGs between two clusters. **F** Kaplan–Meier curves for OS of the two clusters

Consensus clustering analysis identified two clusters based on the GSE10846 dataset of 400 samples (Fig. 1A, B). The accuracy of this classification was confirmed by PCA (Fig. 1C). In total, 3765 DEGs (*p* < 0.05 and |log2FC|≥ 1) were identified, of which 1891 were upregulated and 1874 were downregulated (Additional file 3: Table S3). The top 25 upregulated and downregulated genes are shown in a heatmap (Additional file 8: Fig S1A). Enrichment analysis was performed, which included GO keywords, namely biological process, cellular component, molecular function, and KEGG pathways (Fig. 1D, E, Additional file 4: Table S4) [18]. GO terms related to receptor signaling pathway via JAK-STAT (GO:0007259), receptor signaling pathway via STAT (GO:0097696), nucleosome organization (GO:0034728), DNA replication-dependent chromatin assembly (GO:0006335), glucose metabolic process (GO:0006006), tyrosine phosphorylation of STAT protein (GO:0007260), positive regulation of tyrosine phosphorylation of STAT protein (GO:0042531), hexose metabolic process (GO:0019318), regulation of tyrosine phosphorylation of STAT protein (GO:0042509), muscle system process (GO:0003012), and chromatin remodeling (GO:0006338) are found in the biological process. In the molecular function subontology, the structural constituents of chromatin (GO:0030527), actin filament binding (GO:0051015), and actin-binding (GO:0003779) were significantly enriched. Enrichment of cellular components revealed that the DEGs may be involved in the nucleosome (GO:0000786), cluster of actin-based cell projections (GO:0098862), and brush border (GO:0005903) (Fig. 1D). KEGG pathway enrichment analysis indicated that these DEGs may be involved in the phospholipase D signaling pathway (hsa04072), NET formation (hsa04613), human T-cell leukemia virus 1 infection (hsa05166), Epstein-Barr virus infection (hsa05169), systemic lupus erythematosus (hsa05322), JAK-STAT signaling pathway (hsa04630), MAPK signaling pathway (hsa04010), shigellosis (hsa05131), and alcoholism (hsa05034) (Fig. 1E). Kaplan–Meier survival analysis revealed that the survival probability differed considerably between clusters 1 and 2 and that cluster 2 had a better prognosis than cluster 1 (Fig. 1F).

**Fig. 2:**
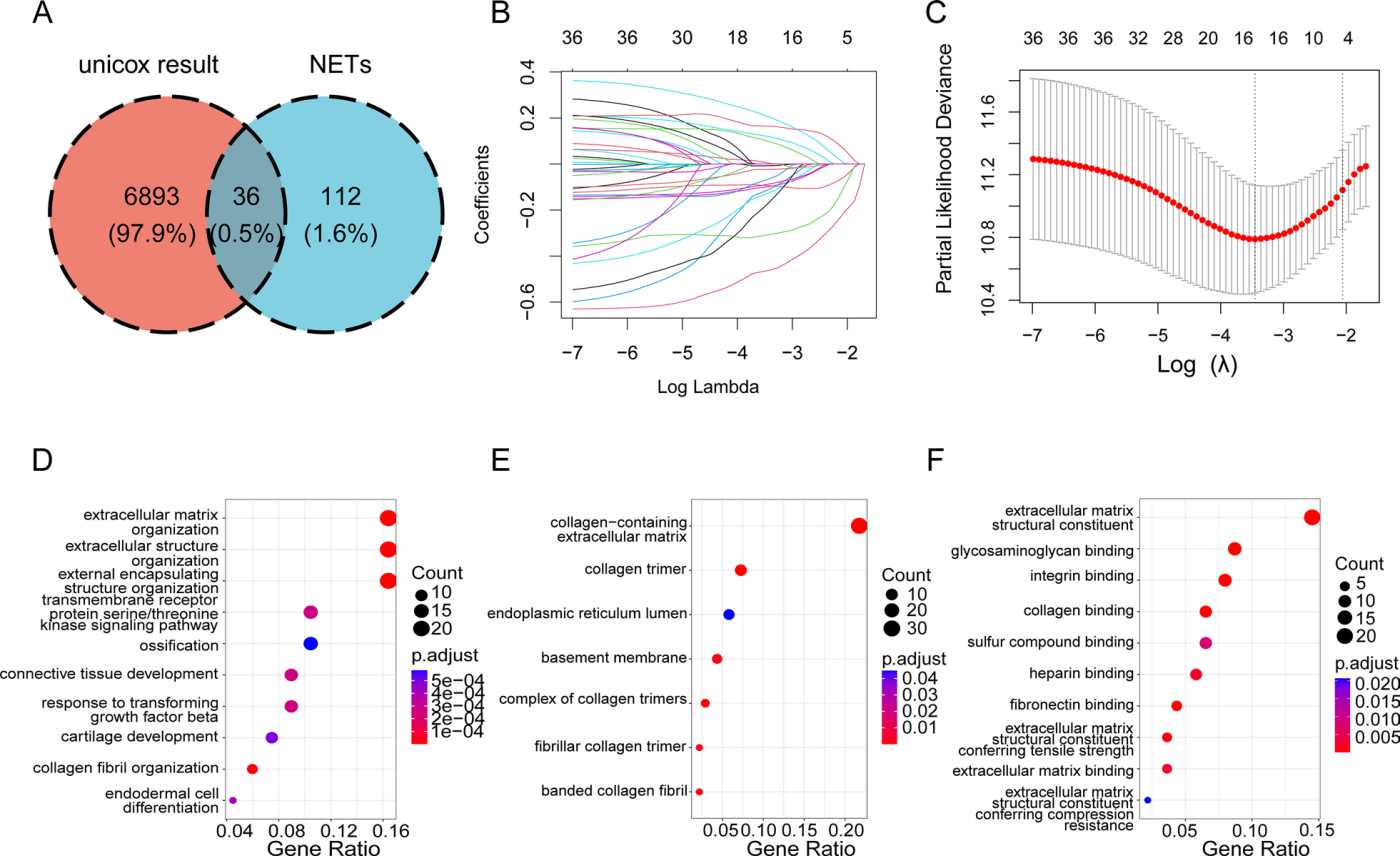
Screening for prognosis-related NRGs and functional identification of DEGs between high- and low-risk groups. **A** Venn diagram analysis showing the overlap of NET sets and univariate Cox results. **B** Establishment of signatures via least absolute shrinkage and selection operator (LASSO) logistic regression analysis. The 36 genes are represented by different colors in the LASSO coefficient profile. **C** Selection of the optimal parameter (lambda) in the LASSO model, and generation of a coefficient profile plot. **D**–**F** Bubble charts depicting GO-enriched DEG items in three functional categories: biological processes (BP, **D**), cell composition (CC, **E**), and molecular function (MF, **F**)

We also identified DEGs associated with overall survival between high- and low-risk patients. A total of 159 DEGs were identified between the two groups, of which 22 were upregulated and 137 were downregulated (*p* < 0.05 and |log2FC|≥ 1). The patients in the two groups showed different gene expression patterns (Additional file 8: Fig S1B). GO term enrichment analysis revealed that the DEGs were mainly involved in the biological processes of extracellular matrix organization (GO:0030198), extracellular structure organization (GO:0043062), and external encapsulating structure organization (GO:0045229) (Fig. 2D). Among the cellular components, collagen-containing extracellular matrix (GO:0062023), collagen trimer (GO:0005581), and collagen trimer complex (GO:0098644) were significantly enriched (Fig. 2E). Enrichment of the molecular function sub-ontology showed that DEGs might be involved in extracellular matrix structural constituents (GO:0005201), glycosaminoglycan binding (GO:0005539), and integrin binding (GO:0005178) (Fig. 2F).

### Identification of survival-related genes

Using univariate Cox regression analysis, survival-related genes were identified based on eight essential OS-related NRGs. We identified genes that were significantly associated with survival in the training dataset (*p* < 0.05) (Additional file 5: Table S5). The Venn diagram showed that 36 NRGs were associated with prognosis (Fig. 2A).

### Recognition of NRG prognostic markers

To avoid potential overfitting, we performed a LASSO Cox regression analysis (Fig. 2B, C). Finally, we selected eight essential OS-related NRGs for modeling based on the following formula: Risk score = [Expression (HIF1A) * (− 0.6495694)] + [Expression (CXCL2) * 0.2840293] + [Expression (CDH1) * (− 0.1415326)] + [Expression (SPP1) * (− 0.1812388)] + [Expression (PPARGCIA) * (0.2010867)] + [Expression (PARVB) * 0.2506490] + [Expression (LYZ) * (− 0.4752112)] + [Expression (S100A9) * 0.1712968].

### Model performance in external datasets

**Fig. 3:**
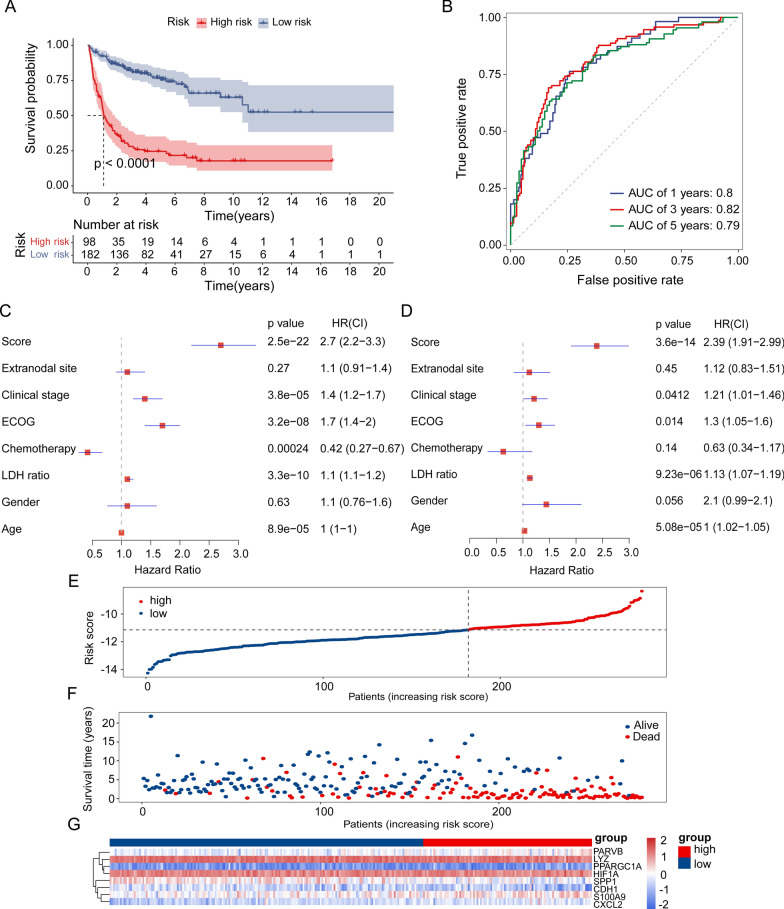
Evaluation of prognostic signature to predict the OS of patients with DLBCL. **A** Patients in the high-risk group had significantly shorter OS than those in the low-risk group. **B** ROC curves for predicting the 1-, 3-, and 5-year survival according to the NRGs score in the training cohort. **C** Univariate Cox regression analysis of the risk scores and clinical parameters. **D** Multivariate Cox regression analysis of the risk scores and cliAaa clinical parameters. **E**, **F** Ranked dot and scatter plots showing the NRGs score distribution and patient survival status. **G** NRG risk model gene expression value

**Fig. 4:**
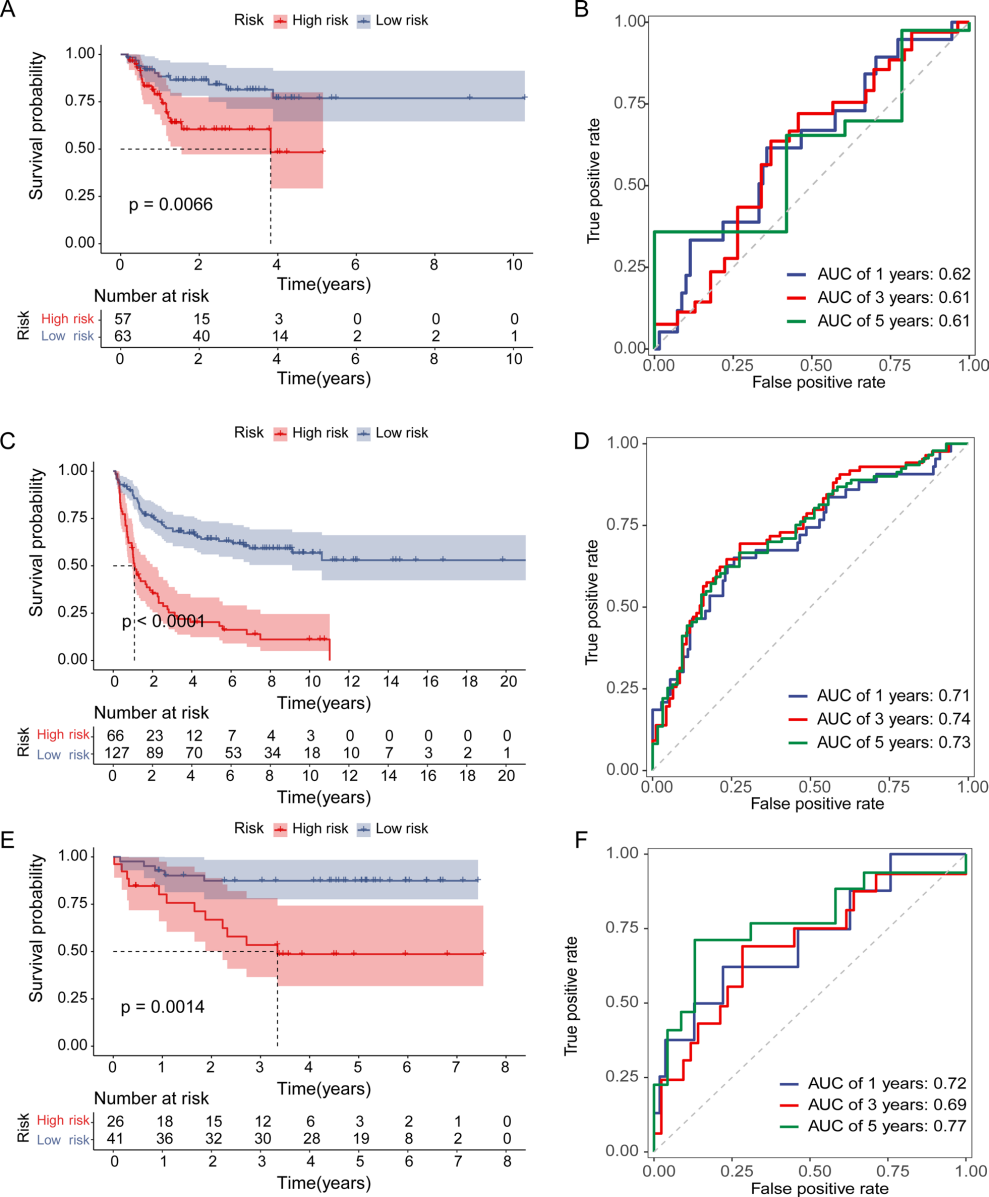
Validation of the NRG prognostic signature on DLBCL cohorts. **A** Kaplan–Meier curve of the prognostic model in the validation cohort. **B** ROC curves for predicting the 1-, 3-, and 5-year survival according to the NRGs score in the validation cohort. **C** Kaplan–Meier curve of the prognostic model in the GSE11318 cohort. **D** ROC curves for predicting the 1-, 3-, and 5-year survival according to the NRGs score in the GSE11318 cohort. **E** Kaplan–Meier curve of the prognostic model in the GSE34171 cohort. **F** ROC curves for predicting the 1-, 3-, and 5-year survival according to the NRGs score in the GSE34171 cohort

According to the optimum cutoff value determined by the R package ‘survminer’ (version 0.4.9), each dataset was automatically split into low- and high-risk groups. Kaplan–Meier analysis showed that patients in the high-risk groups had a significantly poor prognosis than those in the low-risk groups, training set (*p* < 0.0001), validation set (*p* = 0.0066), GSE11318 (*p* < 0.0001), and GSE34171 (*p* = 0.0014) (Figs. 3A and 4A, C, E). We also examined the risk score predictions for 1-, 3-, and 5-year mortality, and the resulting area under the receiver operating characteristic curve (AUC) values in the training dataset were 0.8, 0.82, and 0.79, respectively (Fig. 3B). Additionally, the AUC of the IPI score predictions for 1-, 3-, and 5-year mortality were 0.72, 0.71, and 0.73, respectively (Additional file 9: Fig S2A). Univariate and multifactorial Cox regression analyses were performed to verify the effects of the clinical features and risk scores on patient survival. The risk score was determined to be an independent risk factor (Fig. 3C, D). We then examined the expression of eight NRGs in these patients (Fig. 3G, Additional file 10: Fig S3). In addition, the distribution of sample size and patient survival status revealed a consistent relationship between high risk and high mortality (Fig. 3E–G).

### Construction of a nomogram for prognostic prediction

**Fig. 5:**
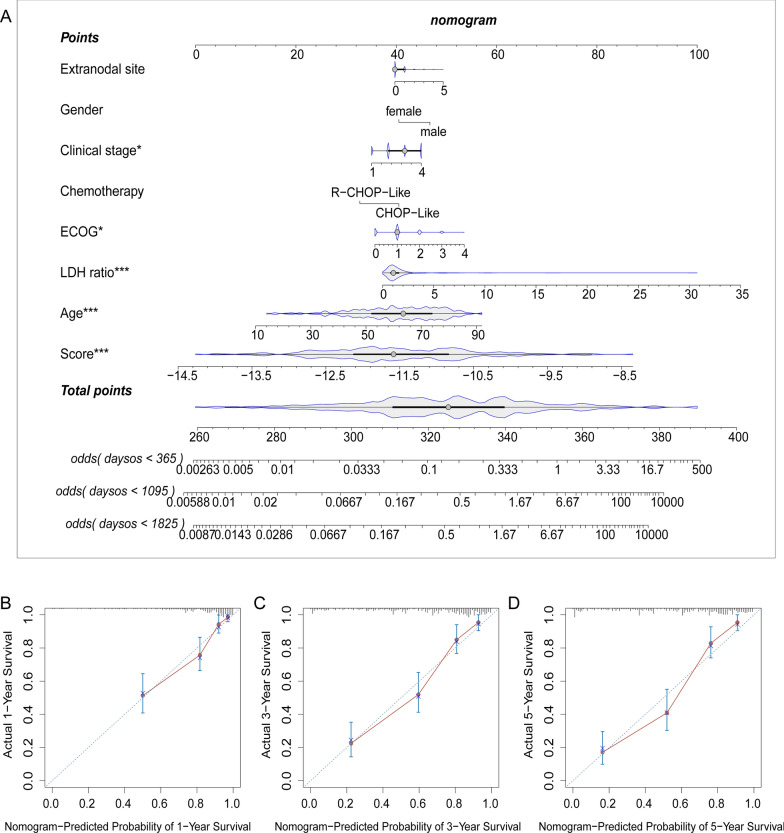
Construction and validation of a nomogram. **A** Nomogram for predicting the 1-, 3-, and 5-year OS of patients with DLBCL in the training cohort. **B**–**D** Calibration curves of the clinicopathologic nomogram predicted and observed 1-, 3-, and 5-year survival of patients with DLBCL

A nomogram was constructed to predict the OS of patients with DLBCL based on the clinical parameters (Fig. 5A). This nomogram was used to assess 1-, 3-, and 5-year OS rates in patients with DLBCL. The calibration curves of this well-established nomogram demonstrated excellent consistency between the actual observations and the predicted values (Fig. 5B–D).

### The immune status of patients in different risk groups

**Fig. 6:**
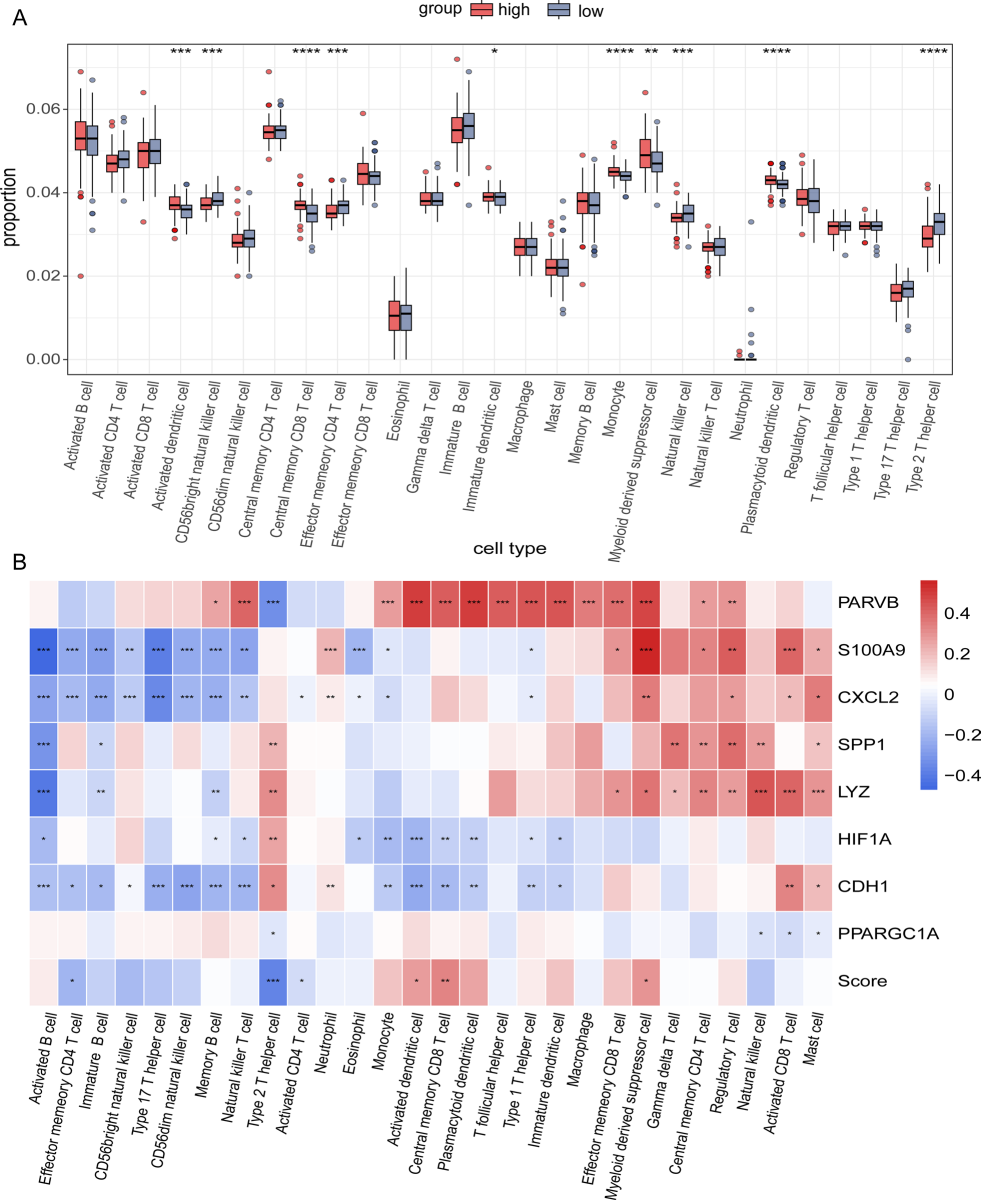
Tumor immune microenvironment analysis of the high- and low-risk groups. **A** Difference between tumor-infiltrating immune cells. The blue box reflects the low-risk group and the red box represents the high-risk group. **B** Heatmap showing Spearman’s correlations between differential immune cells and eight OS-related NRGs. Blue denotes a negative correlation and red denotes a positive correlation. The correlation coefficient increases with the degree of color. *p < 0.05, **p < 0.01, ***p < 0.001

Different immune cell types showed different degrees of infiltration into the tumor microenvironment in the low- and high-risk groups (Additional file 9: Fig S2B, Fig. 6A). Activated dendritic cells, central memory CD8+ T cells, plasmacytoid dendritic cells, myeloid-derived suppressor cells, and monocytes showed higher infiltration levels in the high-risk group than in the low-risk group. In contrast, the numbers of type 2T helper cells, CD56bright natural killer cells, effector memory CD4 T cells, and natural killer cells were higher in the low-risk group (Fig. 6A). In addition, we found that the expression of some NRGs had positive correlations with tumor-infiltrating immune cells, including PARVB, SPP1, and LYZ. In contrast, CDH1 and HIF1A levels were negatively correlated with most tumor-infiltrating immune cells. Meanwhile, a few NRGs, such as S100A9 and CXCL2, were bidirectional (Fig. 6B).

### Analysis of correlations between risk scores and immunological checkpoints

**Fig. 7:**
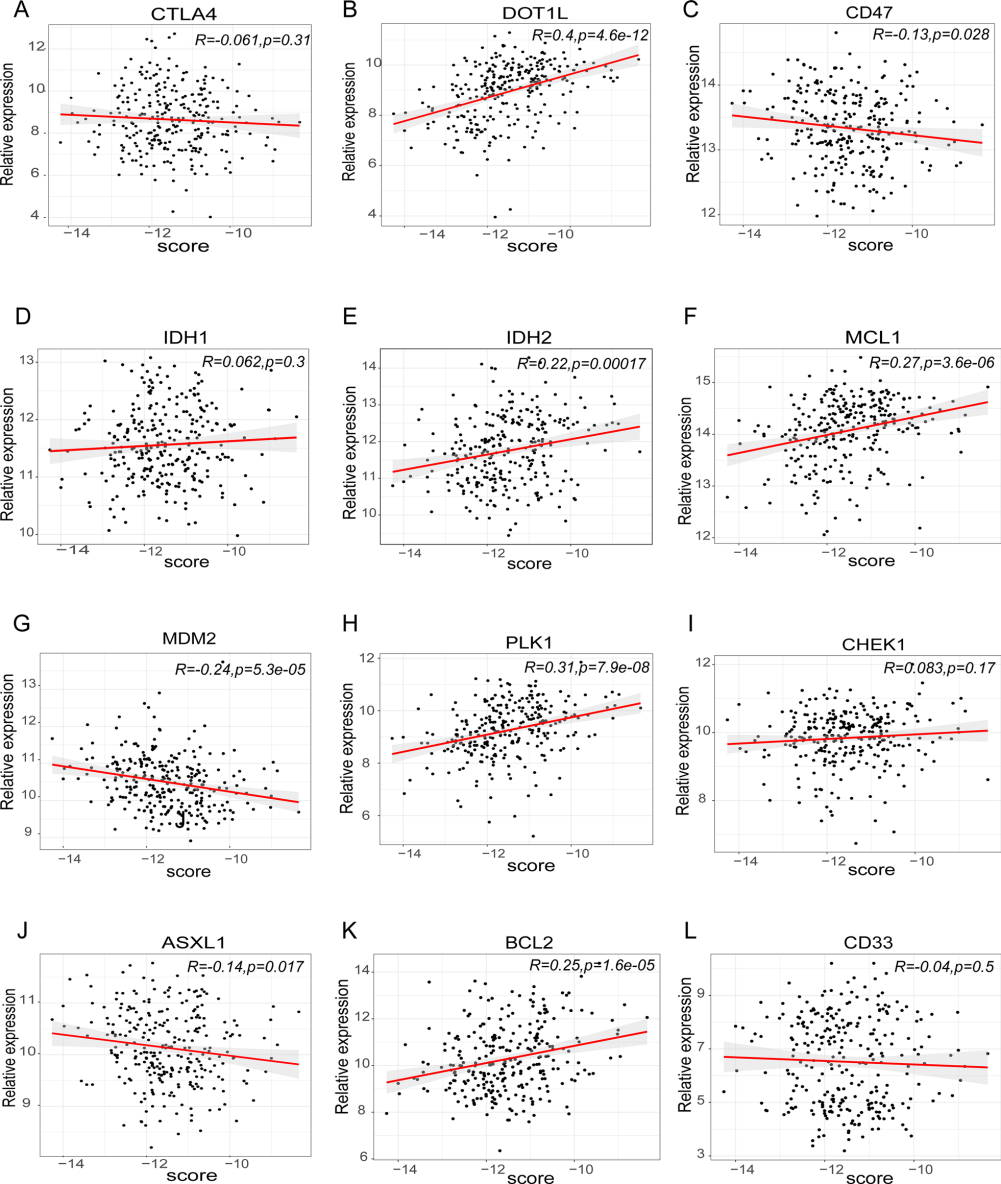
Pearson correlation of the risk scores of the targets of immunotherapy and targeted therapy. **A** CTLA4. **B** DOT1L. **C** CD47. **D** IDH1. **E** IDH2. **F** MCL1. **G** MDM2. **H** PLK1. **I** CHEK1. **J** ASXL1. **K** BCL2. **L** CD33

Immune checkpoint inhibitors were recently discovered and studied in clinical trials for the treatment of patients with DLBCL. According to Pearson correlation analysis, the risk score was significantly and negatively associated with the mRNA expression levels of CD47 (cor = − 0.13, *p* = 0.028), MDM2 (cor = − 0.24, *p* = 5.3e−05), and ASXL1 (cor = − 0.14, *p* = 0.017). In contrast, the risk score was positively correlated with the mRNA expression levels of DOT1L (cor = 0.4, *p* = 4.6e−12), IDH2 (cor = 0.22, *p* = 0.00017), MCL1 (cor = 0.27, *p* = 3.6e−06), PLK1 (cor = 0.31, *p* = 7.9e−08), and BCL2 (cor = 0.25, *p* = 1.6e−05) (Fig. 7). This suggests that targeted therapies against DOT1L, IDH2, MCL1, PLK1, and BCL2 may be able to benefit patients in the high-risk group.

### IC50 analysis

**Fig. 8:**
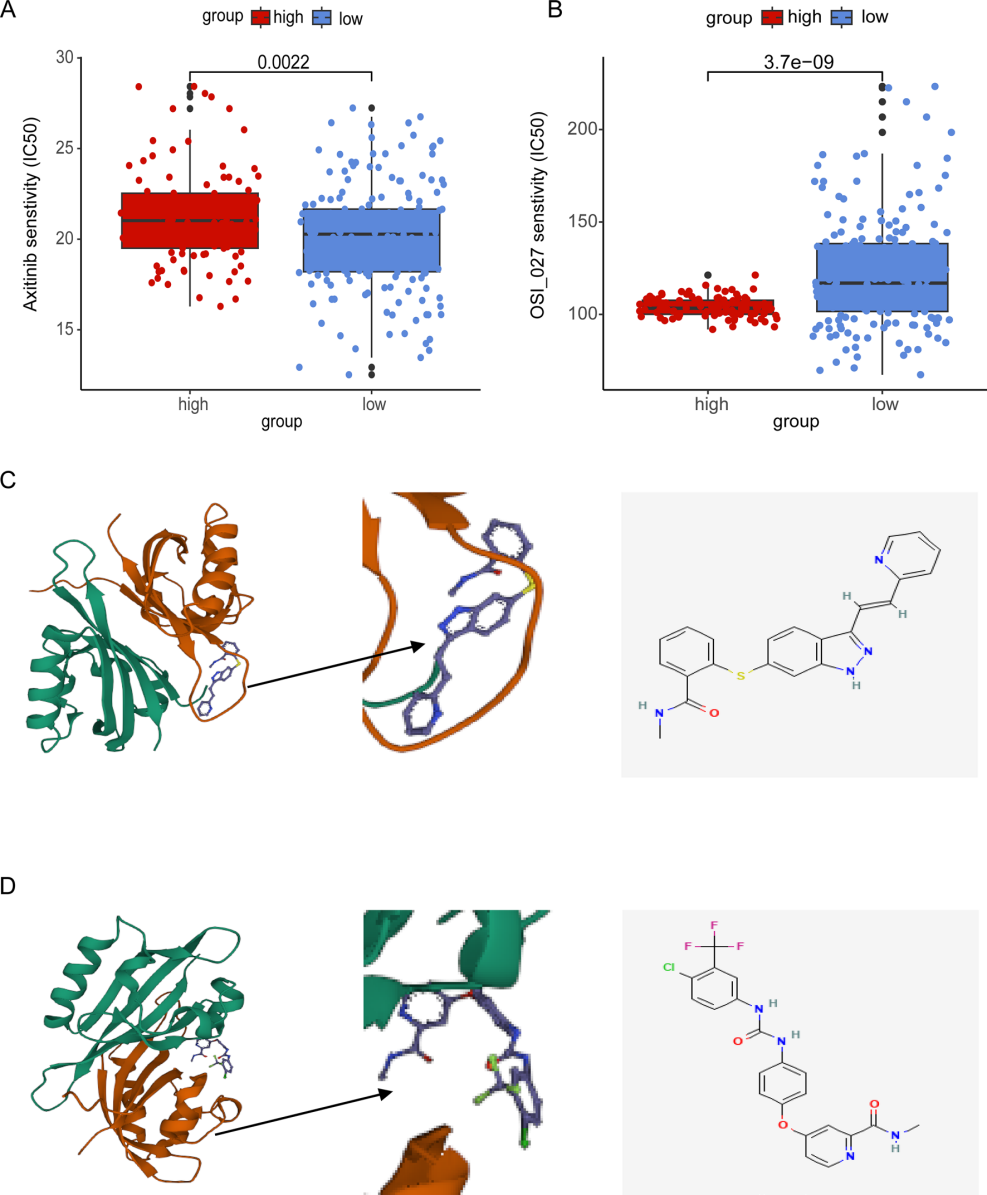
Relationships between risk score and therapeutic sensitivity and molecular docking simulation. **A** Treatment response of axitinib. **B** Treatment response of OSI_027. **C** Left: HIF1A- AXITINIB. Right: Chemical formula of AXITINIB. **D** Left: HIF1A-SORAFENIB. Right: Chemical formula for SORAFENIB

We estimated the IC50 values of 198 drugs for each DLBCL patient between the two groups using the GDSC database. We discovered that the IC50 value of axitinib was higher in the high-risk group than in the low-risk group, whereas that of OSI_027 showed the opposite trend (Fig. 8A, B). This suggests that patients with low NRG scores may respond positively to axitinib, while patients with high NRG scores respond better to OSI_027. Axitinib is an inhibitor of vascular endothelial growth factor receptors (VEGFR), which are associated with tumor angiogenesis. And blockade of the VEGFR receptor pathway inhibits angiogenesis, thereby inhibiting tumor cell proliferation. OSI-027 is a selective and potent dual mTORC1 and mTORC2 inhibitor that induces autophagy in cancer cells. Overall, these results indicated that NRGs were associated with drug sensitivity.

### Drug-gene networks

To explore the potential drugs targeting eight NRGs for the treatment of DLBCL, we searched the DGIdb database, as shown in Additional file 6: Table S6, along with 50 approved therapeutic compounds targeting four genes (HIF1A, SPP1, CDH1, and CCL2). There are 41 relatively abundant potential HIF1A-targeting medications, including nifedipine and piretanide. In addition, we found an association between the SPP1 gene and four potential medicines, including gentamicin and tacrolimus. Lapatinib, erlotinib, and capecitabine can potentially regulate CDH1 gene expression. Finally, the CXCL2 gene may interact with alteplase and deferoxamine. These compounds that target NRGs may be potential DLBCL treatments, but they should be used cautiously until additional mechanisms are investigated (Additional file 6**:** Table S6).

### Molecular docking

Molecular docking was used to screen potential drug candidates and elucidate the molecular mechanisms involved. AutoDock Vina was used for drug-protein molecular docking to screen for optimal potential drug targets. The affinity score was used to measure the merits of a docking process. A high absolute score indicates stronger binding between small molecules and proteins. The molecular docking results showed that all binding energies were negative. The docking scores of potential drugs (Fig. 8C, D) suggest that HIF1A (PDB ID:4H6J) had the strongest binding affinity for axitinib (− 8.5 kcal/mol, |affinity|)\sorafenib (− 8.5 kcal/mol, |affinity|) (Additional file 7: Table S7).

### qPCR results

**Fig. 9:**
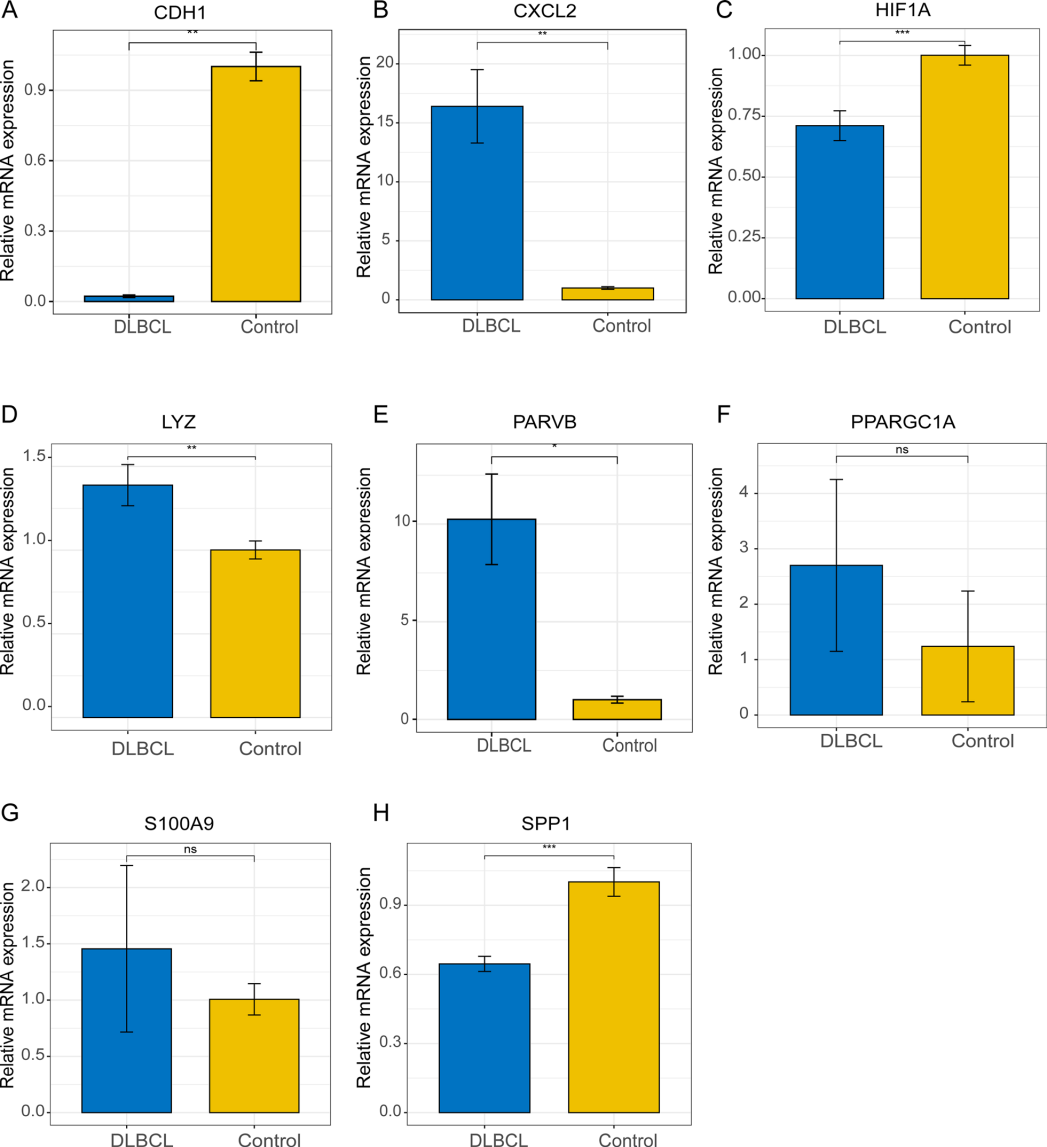
Relative expression of the eight NRGs was assessed by qPCR. **A** CDH1. **B** CXCL2. **C** HIF1A. **D** LYZ. **E** PARVB. **F** PPARGCA1. **G** S100A9. **H** SPP1. *p < 0.05, **p < 0.01, ***p < 0.001

To further validate the expression of the eight NRGs, we performed qPCR on DLBCL cells (OCI-LY3) and human peripheral blood B lymphocytes (IM-9). The levels of CXCL2, LYZ, and PARVB were significantly higher in the DLBCL group (Fig. 9B, D, E). Meanwhile, the control group showed considerably higher levels of mRNA expression of CDH1, HIF1A, and SPP1 (Fig. 9A, C, H). PPARGC1A and S100A9 expression levels did not differ significantly between the two groups (Fig. 9F, G).

## Discussion

DLBCL is one of the most common aggressive malignancies in adults. According to a previous study, the prognosis of a patient may no longer be accurately predicted using IPI scores [27]. Consequently, there is an urgent need to develop reliable biomarkers for prognostic evaluation. In the present study, we focused on the role of NETs in the prognosis of patients with DLBCL. We constructed a predictive risk model based on the eight OS-related NRGs, which was found to be an effective and independent prognostic tool with superior predictive capability compared with the traditional IPI score. Finally, we verified the expression of these eight genes using in vitro qPCR. The experimental results generally agree with our expectations, indicating that the data mining results are credible and have potential research value. In conclusion, our study identified a novel and reliable prognostic biomarker for patients with DLBCL.

Consensus clustering is an unsupervised clustering algorithm that discovers probable groups based on the intrinsic properties of data [16]. Using this method, we identified two molecular subtypes with notably different prognoses. According to KEGG analysis results, DEGs between the two groups were considerably enriched in NETs. This finding supports our hypothesis that NRGs can function as a novel prognostic marker in patients with DLBCL.

To further evaluate the predictive efficacy of the NRGs, we constructed an eight-gene risk model in the training dataset using univariate and LASSO Cox regression analyses. This score served as an independent prognostic marker. The ROC curve showed that the prognostic performance of the model was significantly superior to that of the IPI score. We constructed a nomogram containing the clinical information and prognostic scores of the patients to extend the clinical utility of the model. The calibration curves indicate that the model has an excellent prediction ability. We verified the model in the testing and validation datasets and found that it had excellent predictive performance. To the best of our knowledge, this study is the first to assess the role of NRGs in DLBCL. The NETs risk model is still highly applicable in other solid tumors. Xin et al. constructed a prognostic model based on NETs for hepatocellular carcinoma, and the 1-, 3-, and 5-year AUCs in the training set were 0.836, 0.879, and 0.902, respectively [28]. The NRGs risk model constructed in breast cancer by Zhao et al. also showed good predictive performance, the respective AUCs for the dataset were 0.73, 0.80, and 0.78 [29]. These studies implied that prognostic models based on NETs may be able to have potential prognostic significance in solid tumors.

Genes in the NRG risk model have various functions in the disease. Parvin beta (PARVB) is involved in actin reorganization and focal adhesion, which are associated with cell adhesion, spreading, and motility [30]. Previous studies have demonstrated that PARVB overexpression may promote endogenous growth and metastasis of tongue squamous cell carcinoma by enhancing tumor migration [31]. In urothelial cancer cells, downregulation of PARVB promotes proliferation and migration [32]. In this study, PARVB was associated with a poor prognosis in DLBCL; however, further research is required to determine precisely how it functions. S100 calcium-binding protein A9 (S100A9) is a calcium-binding protein that is mainly produced by neutrophils and monocytes [33]. S100A9 can promote cell proliferation, migration, and invasion in a variety of malignancies [34–36]. C-X-C motif chemokine ligand 2 (CXCL2) is a chemokine generated by activated monocytes and neutrophils. Previous studies showed that compared to normal tissue, DLBCL overexpressed CXCL2 with a fold change of 2.544, and our study reached similar trends [37, 38]. In addition, CXCL2 may be used as a new biomarker for predicting cancer prognosis in specific patients [39]. Secreted phosphoprotein 1 (SPP1) encodes a cytokine that increases the production of interferon-gamma and interleukin-12 [40]. A pan-cancer analysis of SPP1 showed that it promotes immune cell infiltration and that its upregulation is associated with poor prognosis in a variety of cancers [41]. According to Tun et al., SPP1 expression is significantly upregulated in primary central nervous system (CNS) lymphoma; however, its expression in non-CNS DLBCL has not been studied [42]. Lysozyme (LYZ) encodes human lysozyme, which digests the peptidoglycan found in bacterial cell walls. LYZ can be used as a new biomarker in diseases such as acute intracerebral hemorrhage and Graves' orbitopathy [43, 44]. Hypoxia-inducible factor 1 subunit alpha (HIF1A) is a transcription factor that helps cancer cells adapt to hypoxic conditions [45]. Madan et al. found that HIF1A may function as a tumor promoter by degrading the p53 protein and increasing invasive and metastatic activity by binding to five response elements in the p53 promote [46]. Additionally, it is involved in tumor energy metabolism, angiogenesis, and apoptosis, which may help DLBCL cells survival [47].Cadherin 1 (CDH1) codes for calcium-dependent cell adhesion proteins [48, 49]. As an oncogene, its loss of function is thought to contribute to cancer progression through promoting proliferation, invasion, and/or metastasis [50, 51]. The heritable CDH1 mutations are associated with an increased risk of gastric and breast cancers [52]. Alkebsi et al. investigated the expression of CDH1 in DLBCL and non-malignant tissues and showed that CDH1 expression was significantly reduced in lymphomas and our qPCR results also support this conclusion [53]. PPARG coactivator 1 alpha (PPARGC1A) is a transcriptional coactivator that regulates cellular energy metabolism, oxidative phosphorylation, and mitochondrial function. Its expression may be linked to type 2 diabetes and cardiovascular disease [54]. In conclusion, although the roles of some genes in DLBCL have not been investigated, our study confirmed a potential relationship between eight prognostic genes and the prognosis of DLBCL, which will aid future studies in the field.

Most patients with DLBCL can be treated with regular chemotherapy, although up to 40% do not benefit from standard chemotherapy [55]. A recent meta-analysis confirmed the safety and efficacy of immune checkpoint inhibitors in patients with non-Hodgkin lymphoma, suggesting that clinicians may use immune checkpoint inhibitor therapy as an adjunctive therapy [56]. Since DOT1L, IDH2, MCL1, PLK1, and BCL2 were positively correlated with the risk score, patients may benefit from the inhibition of these genes. Conversely, patients may not benefit from blocking CD47, MDM2, or ASXL1 expression. However, larger long-term follow-up trials are required to validate the safety and effectiveness of ICI treatment in patients with DLBCL.

To prevent treatment resistance, it is urgent to identify targeted drugs for DLBCL. The DGIdb database contains 50 FDA-approved target medicines based on eight NRGs. Epoetin alfa acts as an erythropoietin (EPO) and is a growth factor produced in the kidneys that stimulates the production of red blood cells, decreases the number of PRBC transfusions, and does not appear to negatively impact remission duration [57, 58]. Considering the large number of target drugs for HIF1A, we utilized molecular docking to select the best candidates. Interestingly, axitinib and sorafenib showed the strongest binding to HIF1A via hydrogen bond formation. Due to the complexity of cellular signaling, several genes with low expression are also crucial in the development of cancer. In lung cancer, PTEN loss leads to the formation of an immunosuppressive microenvironment which offers resistance to anti-PD-1 therapy [59]. The expression of GPC5 decreased significantly in lung adenocarcinoma tissues, and the low expression of this gene was associated with poor outcomes in lung adenocarcinoma [60]. HIF1A was found to be highly expressed in the low-risk group in our study (*p* = 7e−12). Although the precise mechanism is still unknown, the difference in HIF1A expression between the high-risk and low-risk groups may be related to the two groups' different tumor immune microenvironments. Based on a clinical trial (NCT00071006), axitinib has been tested in patients with myelodysplastic syndromes, and the results showed that two patients with myelodysplastic syndromes had stable disease for 8.3 and 6.2 months, respectively [61]. This enlightens us on the potential efficacy of axitinib as an adjuvant treatment for DLBCL. We will conduct future studies on the specific efficacy of axitinib against HIF1A and explore its intrinsic mechanism.

## Conclusions

We identified the NRGs associated with prognosis and developed an eight-gene prognostic model. The model provides a prognostic score that is independent of other factors. Additionally, we validated the robustness of the model using multiple methods and obtained satisfactory results. Our study not only analyzed the predictive performance of the risk model but also screened prospective treatment drugs.

## Limitations

Our study has some limitations. First, the GEO provides only a limited amount of information on clinical features and may not include other clinical parameters. Second, the data were obtained from retrospective studies, which may have been influenced by selection bias. Prospective investigations are required to confirm our findings. Further research is needed to determine how these eight OS-related genes function in DLBCL.

### Supplementary Information

**Additional file 1: Table S1a.** Clinical Information of GSE10846. **Table S1b.** Clinical Information of GSE11318. **Table S1c.** Clinical Information of GSE34171.

**Additional file 2: Table S2.** Information of 148 NET-related genes (NRGs) collected in this research.

**Additional file 3: Table S3.** Differentially expressed genes of the training dataset.

**Additional file 4: Table S4a.** Gene Ontology (GO) terms of the DEGs. **Table S4b.** Kyoto Encyclopedia of Genes and Genomes (KEGG) of the DEGs.

**Additional file 5: Table S5a.** Survival-related genes in the training dataset. **Table S5b.** Survival-related genes in the training dataset. (*p* < 0.05). **Table S5c.** Prognosis related NRGs.

**Additional file 6: Table S6.** Prediction of potential therapeutic drugs.

**Additional file 7: Table S7.** Molecular docking analysis of HIF1A.

**Additional file 8: Fig. S1.** The heatmap of DEGs. (A) Heatmap of DEGs between the two clusters. (B) Heatmap of DEGs between the high-risk and low-risk groups.

**Additional file 9: Fig. S2.** (A) ROC curves for predicting the 1-, 3-, and 5-year survival according to the IPI score in the training cohort. (B) The tumor microenvironment in the low- and high-risk groups.

**Additional file 10: Fig. S3.** The relative gene expression between the high- and low- risk group in the training dataset.

## Data Availability

The datasets generated during or analyzed during the current study are publicly available.
