# Supplementary material for: A novel NET-related gene signature for predicting DLBCL prognosis
Source: J Transl Med. 2023 Sep 16;21:630. doi: 10.1186/s12967-023-04494-9 (PMC10504796; doi:10.1186/s12967-023-04494-9)
Supplement: Supplementary file 6 — Additional file 6: Table S6. Prediction of potential therapeutic drugs. [file 12967_2023_4494_MOESM6_ESM.docx]

**Additional file 6 : Table S6. Prediction of potential therapeutic drugs.**

| **Compound** | **Gene** | **Source** | **PubChem ID** |
| --- | --- | --- | --- |
| Phenoxybenzamine Hydrochloride | HIF1A | DTC | 5284441 |
| Nifedipine | HIF1A | DTC | 4485 |
| Piretanide | HIF1A | DTC | 4489 |
| Epinephrine | HIF1A | DTC | 5816 |
| Epinephrine Bitartrate | HIF1A | DTC | 5815 |
| Axitinib | HIF1A | PharmGKB | 6450551 |
| Triamterene | HIF1A | DTC | 5546 |
| Dequalinium | HIF1A | DTC | 2993 |
| Benzbromarone | HIF1A | DTC | 2333 |
| Vincristine Sulfate | HIF1A | DTC | 249332 |
| Inamrinone | HIF1A | DTC | 3698 |
| Nitroglycerin | HIF1A | My Cancer Genome Clinical Trial | 4510 |
| Amcinonide | HIF1A | DTC | 443958 |
| Tretinoin | HIF1A | DTC | 444795 |
| Desoximetasone | HIF1A | DTC | 5311067 |
| Cycloserine | HIF1A | DTC | 6234 |
| Oxytetracycline | HIF1A | DTC | 54675779 |
| Diclofenac Sodium | HIF1A | DTC | 5018304 |
| Niclosamide | HIF1A | DTC | 4477 |
| Isoetharine Mesylate | HIF1A | DTC | 23702 |
| Loratadine | HIF1A | DTC | 3957 |
| Hydroquinone | HIF1A | DTC | 785 |
| Oxatomide | HIF1A | DTC | 4615 |
| Isoproterenol | HIF1A | DTC | 3779 |
| Mefenamic Acid | HIF1A | DTC | 4044 |
| Dopamine | HIF1A | DTC | 681 |
| Tolfenamic Acid | HIF1A | DTC | 610479 |
| Sulfasalazine | HIF1A | DTC | 5339 |
| Epoetin Alfa | HIF1A | NCI | 92043599 |
| Ethamsylate | HIF1A | DTC | 17506 |
| Flufenamic Acid | HIF1A | DTC | 3371 |
| Norepinephrine Bitartrate | HIF1A | DTC | 3047796 |
| Clotrimazole | HIF1A | DTC | 2812 |
| Sorafenib | HIF1A | PharmGKB | 216239 |
| Promazine | HIF1A | DTC | 4926 |
| Levonordefrin | HIF1A | DTC | 164739 |
| Pimozide | HIF1A | DTC | 16362 |
| Deferoxamine | HIF1A | NCI | 2973 |
| Noscapine | HIF1A | Tdg Clinical Trial | 275196 |
| Hydrocortisone | HIF1A | DTC | 5754 |
| Topotecan Hydrochloride | HIF1A | DTC | 60699 |
| Alteplase | SPP1 | NCI | none |
| Gentamicin | SPP1 | NCI | 3467 |
| Tacrolimus | SPP1 | NCI | 445643 |
| Calcitonin | SPP1 | NCI | 118984394 |
| Lapatinib | CDH1 | JAX-CKB | 208908 |
| Erlotinib | CDH1 | JAX-CKB | 176870 |
| Capecitabine | CDH1 | JAX-CKB | 60953 |
| Alteplase | CXCL2 | NCI | none |
| Deferoxamine | CXCL2 | NCI | 2973 |
