# Supplementary material for: A novel NET-related gene signature for predicting DLBCL prognosis
Source: J Transl Med. 2023 Sep 16;21:630. doi: 10.1186/s12967-023-04494-9 (PMC10504796; doi:10.1186/s12967-023-04494-9)
Supplement: Supplementary file 7 — Additional file 7: Table S7. Molecular docking analysis of HIF1A. [file 12967_2023_4494_MOESM7_ESM.docx]

**Additional file 7: Table S7. Molecular docking analysis of HIF1A.**

| **Target** | **Compound** | **Energy (kcal/Mol)** |
| --- | --- | --- |
| HIF1A | Phenoxybenzamine Hydrochloride | -5.6 |
| HIF1A | Nifedipine | -7.7 |
| HIF1A | Piretanide | -6.5 |
| HIF1A | Epinephrine | -5.0 |
| HIF1A | Epinephrine Bitartrate | -4.9 |
| HIF1A | Axitinib | -8.5 |
| HIF1A | Triamterene | -6.6 |
| HIF1A | Dequalinium | -6.1 |
| HIF1A | Benzbromarone | -6.6 |
| HIF1A | Vincristine Sulfate | -7.2 |
| HIF1A | Inamrinone | -5.6 |
| HIF1A | Nitroglycerin | -5.4 |
| HIF1A | Amcinonide | -7.4 |
| HIF1A | Tretinoin | -6.5 |
| HIF1A | Desoximetasone | -7.9 |
| HIF1A | Cycloserine | -3.9 |
| HIF1A | Oxytetracycline | -6.7 |
| HIF1A | Diclofenac Sodium | -6.1 |
| HIF1A | Niclosamide | -6.4 |
| HIF1A | Isoetharine Mesylate | -5.5 |
| HIF1A | Loratadine | -7.6 |
| HIF1A | Hydroquinone | -3.9 |
| HIF1A | Oxatomide | -7.1 |
| HIF1A | Isoproterenol | -5.5 |
| HIF1A | Mefenamic Acid | -6.2 |
| HIF1A | Dopamine | -4.6 |
| HIF1A | Tolfenamic Acid | -6.1 |
| HIF1A | Sulfasalazine | -7.5 |
| HIF1A | Epoetin Alfa | -5.9 |
| HIF1A | Ethamsylate | -5.2 |
| HIF1A | Flufenamic Acid | -6.9 |
| HIF1A | Norepinephrine Bitartrate | -7.1 |
| HIF1A | Clotrimazole | -7.5 |
| HIF1A | Sorafenib | -8.5 |
| HIF1A | Promazine | -5.4 |
| HIF1A | Levonordefrin | -5.1 |
| HIF1A | Pimozide | -7.2 |
| HIF1A | Deferoxamine | -6.5 |
| HIF1A | Noscapine | -5.7 |
| HIF1A | Hydrocortisone | -6.7 |
| HIF1A | Topotecan Hydrochloride | -5.3 |
