# Supplementary figures and images for: A novel NET-related gene signature for predicting DLBCL prognosis
Source: J Transl Med. 2023 Sep 16;21:630. doi: 10.1186/s12967-023-04494-9 (PMC10504796; doi:10.1186/s12967-023-04494-9)

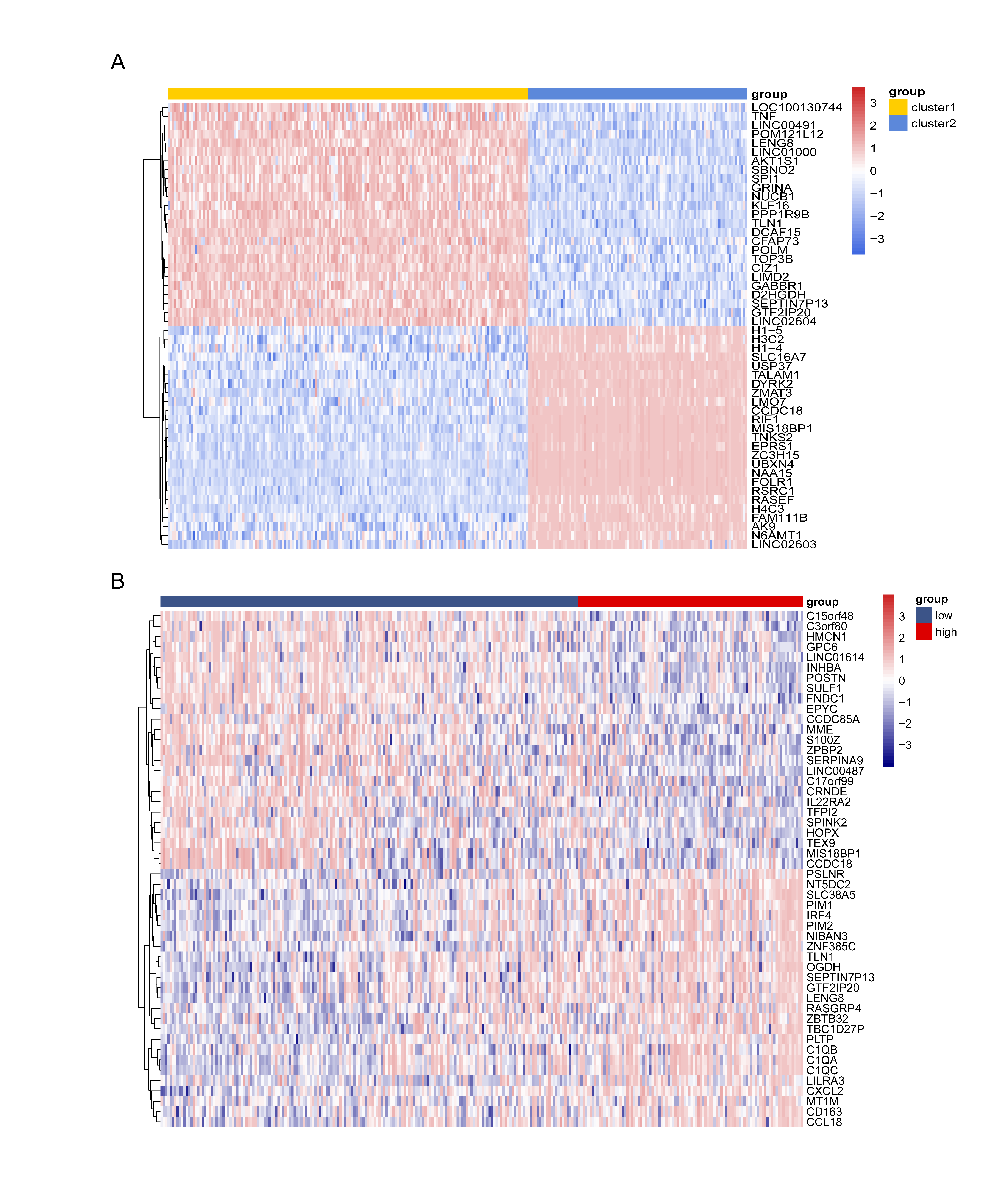

Supplement: Supplementary file 8 — Additional file 8: Fig. S1. The heatmap of DEGs. (A) Heatmap of DEGs between the two clusters. (B) Heatmap of DEGs between the high-risk and low-risk groups. [file 12967_2023_4494_MOESM8_ESM.tif]

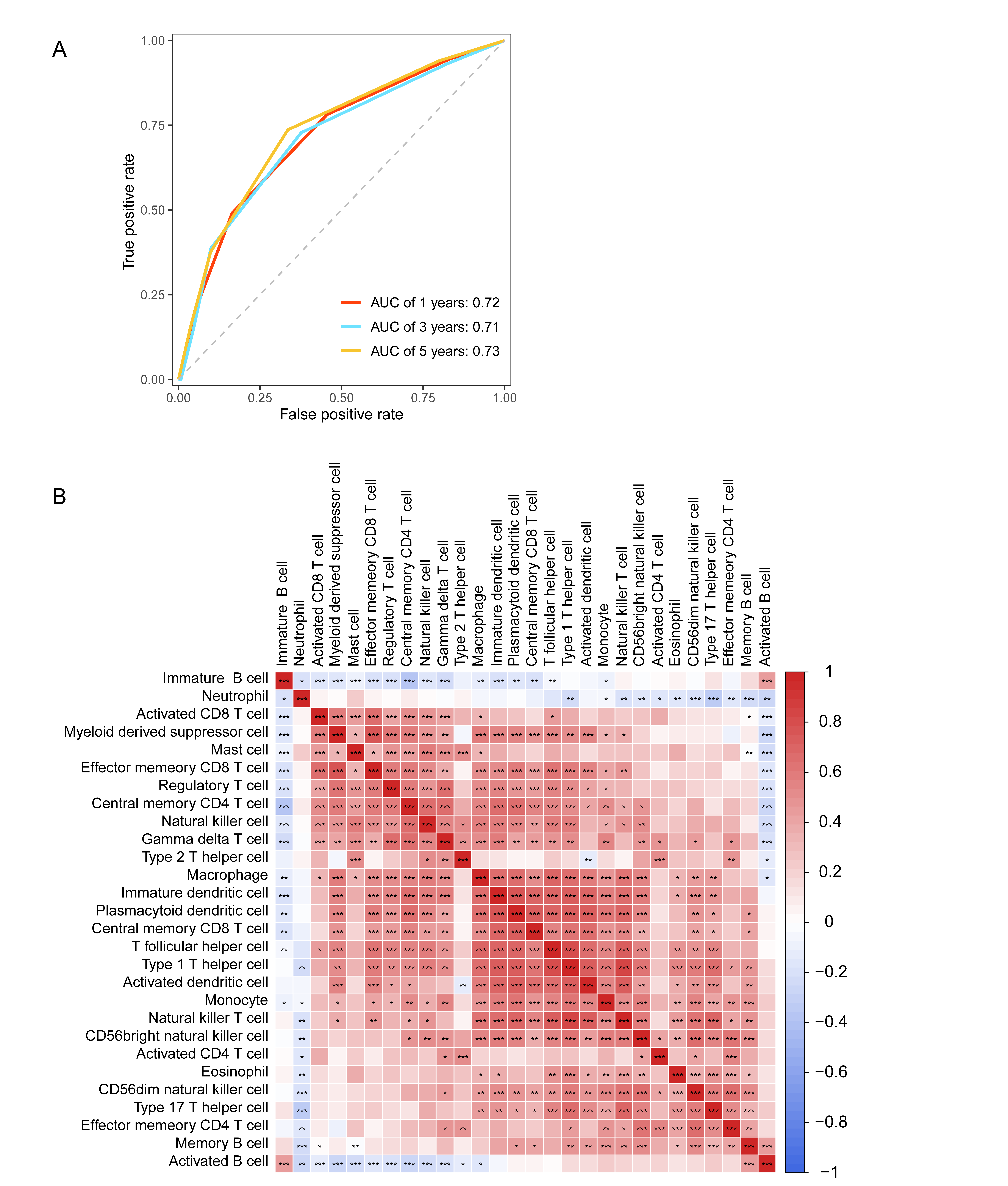

Supplement: Supplementary file 9 — Additional file 9: Fig. S2. (A) ROC curves for predicting the 1-, 3-, and 5-year survival according to the IPI score in the training cohort. (B) The tumor microenvironment in the low- and high-risk groups. [file 12967_2023_4494_MOESM9_ESM.tif]

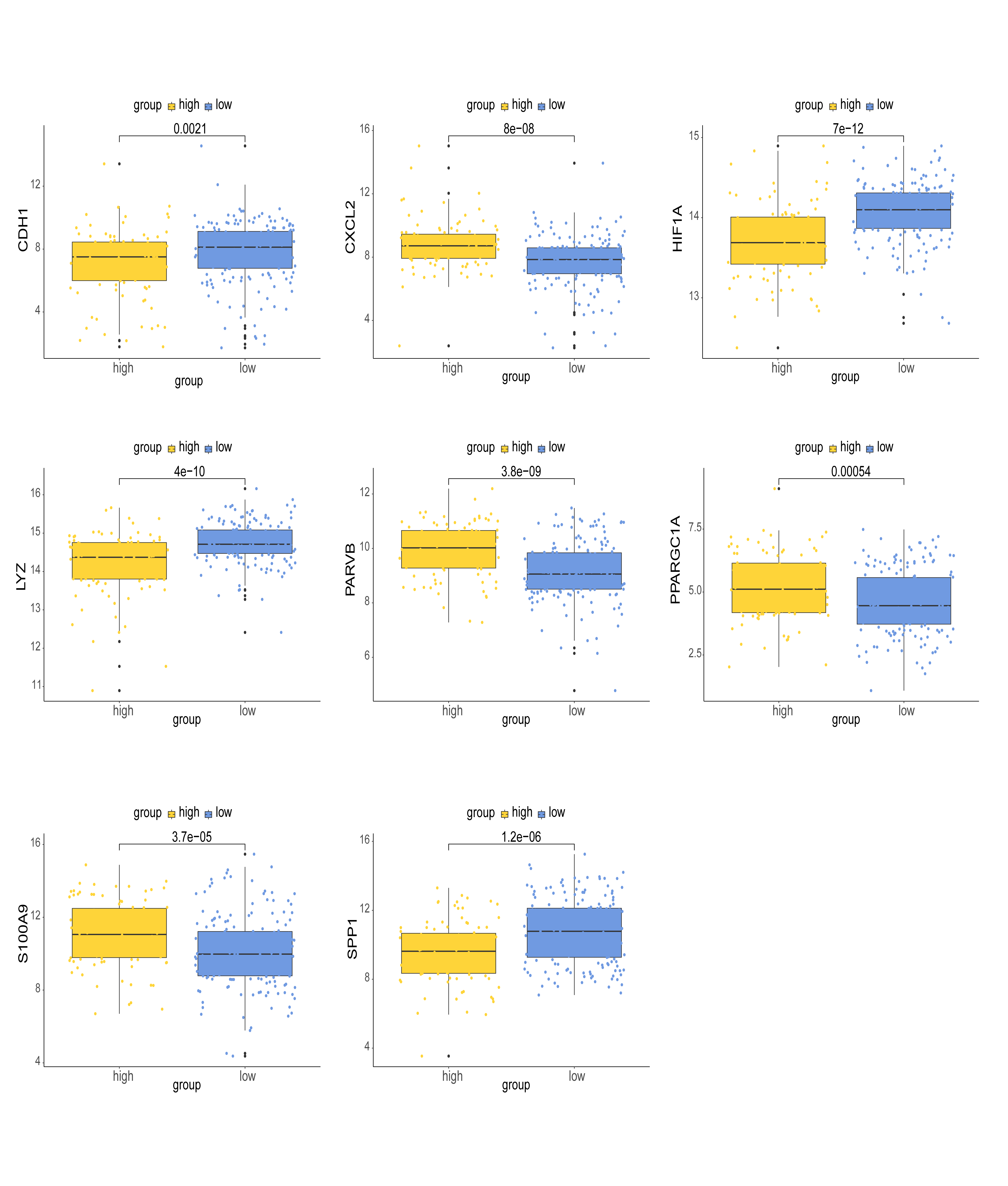

Supplement: Supplementary file 10 — Additional file 10: Fig. S3. The relative gene expression between the high- and low- risk group in the training dataset. [file 12967_2023_4494_MOESM10_ESM.tif]
